# Propensity score matching after multiple imputation when a confounder has missing data

**DOI:** 10.1002/sim.9658

**Published:** 2023-01-25

**Authors:** Corentin Ségalas, Clémence Leyrat, James R. Carpenter, Elizabeth Williamson

**Affiliations:** ^1^ Department of Medical Statistics London School of Hygiene and Tropical Medicine London UK; ^2^ Université Paris‐Cité Centre of Epidemiology and Statistics (CRESS) Inserm Paris France; ^3^ MRC Clinical Trials Unit at UCL UCL London UK

**Keywords:** confounding, missing data, multiple imputation, propensity score matching

## Abstract

One of the main challenges when using observational data for causal inference is the presence of confounding. A classic approach to account for confounding is the use of propensity score techniques that provide consistent estimators of the causal treatment effect under four common identifiability assumptions for causal effects, including that of no unmeasured confounding. Propensity score matching is a very popular approach which, in its simplest form, involves matching each treated patient to an untreated patient with a similar estimated propensity score, that is, probability of receiving the treatment. The treatment effect can then be estimated by comparing treated and untreated patients within the matched dataset. When missing data arises, a popular approach is to apply multiple imputation to handle the missingness. The combination of propensity score matching and multiple imputation is increasingly applied in practice. However, in this article we demonstrate that combining multiple imputation and propensity score matching can lead to over‐coverage of the confidence interval for the treatment effect estimate. We explore the cause of this over‐coverage and we evaluate, in this context, the performance of a correction to Rubin's rules for multiple imputation proposed by finding that this correction removes the over‐coverage.

## 1 INTRODUCTION

While randomized controlled trials are considered the gold standard for causal inference in the medical sciences, they are not always feasible.^1^ Often, data from observational data must be used to address causal questions.^2^ However, observational studies are prone to confounding which means that unadjusted analyses would lead to bias.^3^, ^4^ Various statistical methods to adjust for observed confounding exist, for example, multivariable regression. However, propensity score methods are an increasingly popular approach. The propensity score is a balancing score, which means that, at any particular value of the propensity score, the distribution of the baseline covariates is the same among treated and untreated patients. Under four key assumptions,^5^ including that of no unmeasured confounding, a range of propensity score methods aim to achieve balance of observed confounders between treatment groups, with the view of mimicking randomization.

Despite some criticism in the literature,^6^ the propensity score matching method is the most widely used in practice in medical research.^7^, ^8^, ^9^, ^10^ Many different implementations of propensity score matching exist depending on: the matching algorithm, greedy or optimal, the metric used,^11^, ^12^ the presence and size of a caliper that limits the difference between propensity scores of a matched pair,^13^, ^14^, ^15^ the number of non‐treated patients matched to each treated patient^16^ and the presence or absence of replacement in the sampling.^12^, ^17^ Based on the distance between their estimated propensity scores, treated patients are matched to non‐treated patients in order to create a matched sample in which the two treatment groups have similar characteristics. Whether and how to take account of the matching in the analysis has been contested in the literature.^7^, ^18^

In the following, we consider the setting of a cohort study, with potential confounders measured at the study baseline, a treatment of interest assessed at study baseline and an outcome of interest assessed during follow‐up.

An issue for propensity score matching, as for any other adjustment method, is the presence of missing data on confounders. Multiple imputation is a powerful and increasingly popular approach to handle missing data. M completed datasets are built by drawing missing values from their posterior predictive distribution. Treatment effect estimates are then obtained for each completed dataset and are averaged into a single treatment effect estimate. Rubin^19^ proposed rules to obtain a variance effect estimate that correctly accounts for the additional variability caused by the missing data.

Multiple imputation has been proposed in combination with propensity score analysis. Leyrat et al^20^ discuss challenges in applying multiple imputation within propensity score analysis in general. Here, we focus on specific issues encountered when applying propensity score matching. The most notable feature of propensity score matching, in contrast to other propensity score approaches, is that a portion of the data used to generate the estimated propensity scores is discarded in the final analysis. This means that the sample used to estimate the treatment effect will be only a subset of the sample used to fit the imputation models. In the context of multiple imputation for measurement error, Reiter^21^ noticed that if some patients contributed to the imputation model but not to the analysis model, Rubin's rules could lead to inflated variances and over‐coverage. This reflects a general phenomenon of over‐coverage arising when some information is available to the imputer that is not available to the subsequent analyst. For example, if an imputation model correctly omits an interaction that is allowed for in the analysis model, no bias is induced but over‐coverage occurs.^22^

Reiter proposed a new approach, adding a bootstrapping step to Rubin's rules, leading to confidence intervals with correct coverage in the measurement error setting. Parallels between Reiter's setting and the current one ‐ namely the use of data to inform imputation models which is then discarded prior to fitting the substantive model ‐ raise the question of whether similar over‐coverage occurs when using Rubin's rules to obtain variance estimates following multiple imputation in the context of propensity score matching. Therefore, our objectives are to establish whether discarding unmatched patients in propensity score matching, following multiple imputation, does lead to over‐coverage of the confidence interval for the treatment effect estimate and if, as we expect, we do observe this phenomenon, to evaluate the performance of Reiter's correction in this context.

This article is structured as follows. Section 2 introduces key statistical concepts and methodology. Section 3 presents a simulation study assessing the statistical properties of confidence intervals obtained by applying Rubin's rules, following the application of multiple imputation and propensity score matching, and by applying Reiter's proposed correction. An illustrative example estimating the effect of age on the probability of receiving surgery for lung cancer in a UK cohort study is performed in Section 4. Finally, some concluding remarks are given in Section 5.

## 2 METHODS

Suppose our fully observed data consists of, for each patient i=1,…,N, a binary outcome $y_{i}$, a binary treatment $z_{i}$ and some baseline covariates $x_{i}^{⊤}=(x_{1},\ldots ,x_{p})$, all potential confounders.

### 2.1 Estimands

Two common estimands of interest are the average treatment effect (ATE) and the average treatment effect on the treated (ATT). These are defined as

$$\text{ATE}=E(y_{i}^{1})-E(y_{i}^{0}),$$

$$\text{ATT}=E(y_{i}^{1}|z_{i}=1)-E(y_{i}^{0}|z_{i}=1),$$

where $y_{i}^{0}$ and $y_{i}^{1}$ are the values of the outcome for patient i if patient i were not treated and if patient i were treated, respectively. In the counterfactual framework,^23^ $y^{0}$ and $y^{1}$ are the two potential outcomes, only one is observed and the other is called the counterfactual. One can only observe $y_{i}=z_{i}y_{i}^{1}+(1-z_{i})y_{i}^{0}$ because it is impossible to observe both $y_{i}^{0}$ and $y_{i}^{1}$ for a same patient i. Therefore, the ATE and the ATT are not directly computable. Here, we focus on the ATT throughout since that is often the estimand of interest in propensity score matching.

### 2.2 Assumptions

We make the following standard causal inference assumptions. First, we make the stable unit treatment value assumption (SUTVA),^24^ which states that the two potential outcomes $y_{i}^{0}$ and $y_{i}^{1}$ of one patient i cannot be influenced by the treatment of another patient. Secondly, we assume consistency,^25^ which states that for each subject, the potential outcome under the observed treatment exactly matches the observed outcome (ie, if $z_{i}=1$ then $y_{i}=y_{i}^{1}$ and if $z_{i}=0$ then $y_{i}=y_{i}^{0}$). Third, we assume positivity,^26^ which states that each patient has a non‐null probability of receiving the treatment and of not receiving the treatment, that is, $0<p(z_{i}=1|x_{i})<1$. And finally, we assume ignorability^5^ which states that $y^{0},y^{1}\;⊥\;z|x$ which implies that there are no unmeasured confounders.

### 2.3 Propensity score matching and treatment effect estimation

The propensity score was introduced by Rosenbaum and Rubin^5^ and is defined as the probability that a patient i receives treatment conditional on the patient's baseline covariates, $ps_{i}=P(z_{i}=1|x_{i})$. Since this probability is unknown, it is estimated from the data, often using a logistic regression model for observed treatment. Alternatively, more data‐adaptive modeling strategies may be used.^27^

Due to the balancing property of the propensity score, under the assumptions detailed in Section 2.2, 1:1 matching on the estimated propensity score allows a consistent estimator $\hat{\theta }$ of the ATT to be directly computed by comparing the outcomes y between treated and untreated patients. For binary outcomes, while we have defined the ATT as a difference in means, leading to a causal risk difference, analogous definitions on different scales exist (risk ratio, odds ratio, etc.). The variance estimate can be modified to take into account the matched nature of the data^7^ or to take into account the estimation of the propensity scores.^28^ Bootstrap approaches have also been proposed^17^ in this context.

### 2.4 Combining propensity score matching and multiple imputation

Problems arise when there are missing data in at least one of the baseline covariates so that the propensity score is not directly estimable. In this case, a widely used approach is based on the combination of propensity score matching with multiple imputation.^19^ Multiple imputation samples from the posterior distribution of the missing data conditional on the observed data to impute missing values. Typically this is done under the missing at random assumption. The most commonly used implementation of multiple imputation is based on the chained equation method.^29^, ^30^ Implementations of this approach are available in most standard software, for example in the r package mice by van Buuren and Groothuis‐Oudshoorn.^31^

By imputing multiple times, $nb_{imp}$ imputed datasets are created. For each of the $nb_{imp}$ imputed datasets, a propensity score model can be estimated. From there, to obtain an estimate of the treatment effect using propensity score matching, different approaches can be used, notably what have been termed the *within* and *across* approaches.^20^ In the within approach, the matching is done separately for each one of the $nb_{imp}$ imputed datasets leading to $nb_{imp}$ matched datasets and $nb_{imp}$ treatment effect estimates that are aggregated into one estimate.^19^ In the across approach, the propensity scores are averaged over all $nb_{imp}$ imputed datasets and the matching is done from this averaged propensity score leading directly to one treatment effect estimate only. Following initial debate of these two approaches,^32^, ^33^, ^34^, ^35^, ^36^ Leyrat et al^20^ demonstrated that only the within approach could lead to consistent estimates, subsequently confirmed by other authors.^37^, ^38^ Therefore, we adopt the within approach.

The within approach leads to $nb_{imp}$ estimated treatment effects, ${\hat{\theta }}_{k}$, $k=1,\ldots ,nb_{imp}$, which can be aggregated using Rubin's rules^19^ as follows:

$$\hat{\theta }=\frac{1}{nb_{imp}}\overset{nb_{imp}}{\underset{k=1}{\sum }}{\hat{\theta }}_{k},$$

$$\hat{Var}(\hat{\theta })=W+\left(1+\frac{1}{nb_{imp}}\right)B,$$

where W is the within imputation variance and B the between imputation variance

$$W=\frac{1}{nb_{imp}}\overset{nb_{imp}}{\underset{k=1}{\sum }}\hat{Var}({\hat{\theta }}_{k}),$$

$$B=\frac{1}{nb_{imp}-1}\overset{nb_{imp}}{\underset{k=1}{\sum }}{\left({\hat{\theta }}_{k}-\hat{\theta }\right)}^{2}.$$

### 2.5 Reiter's rules

In the context of multiple imputation for measurement error, Reiter^21^ proposed a modification of Rubin's rules to combine estimates across imputed datasets in scenarios where some of the patients used for imputation are not used for further analysis. In classic multiple imputation, a parameter draw computed from the imputation model is used to generate a single imputed dataset. This operation is repeated $nb_{imp}$ times, resulting in $nb_{imp}$ completed datasets each corresponding to a different parameter draw. Reiter^21^ proposed generating not one but $nb_{rep}$ datasets from the same parameter draw and to repeat this $nb_{imp}$ times leading to a total of $nb_{imp}\times nb_{rep}$ imputed datasets. The treatment effect estimates ${\hat{\theta }}_{k,j}$ for $k=1,\ldots ,nb_{imp}$ and $j=1,\ldots ,nb_{rep}$ are aggregated and the variance is estimated using the following formulae:

$$\hat{\theta }=\frac{1}{nb_{imp}nb_{rep}}\overset{nb_{imp}}{\underset{k=1}{\sum }}\overset{nb_{rep}}{\underset{j=1}{\sum }}{\hat{\theta }}_{k,j}=\frac{1}{nb_{imp}}\overset{nb_{imp}}{\underset{k=1}{\sum }}{\hat{\theta }}_{k}\;\text{where}\;{\hat{\theta }}_{k}=\frac{1}{nb_{rep}}\overset{nb_{rep}}{\underset{j=1}{\sum }}{\hat{\theta }}_{k,j},$$

$$\hat{Var}(\hat{\theta })=\tilde{W}+\left(1+\frac{1}{nb_{imp}}\right)\tilde{B}-\left(1+\frac{1}{nb_{rep}}\right)U,$$

where

$$\tilde{W}=\frac{1}{nb_{imp}nb_{rep}}\overset{nb_{imp}}{\underset{k=1}{\sum }}\overset{nb_{rep}}{\underset{j=1}{\sum }}\hat{Var}({\hat{\theta }}_{k,j}),$$

$$\tilde{B}=\frac{1}{nb_{imp}-1}\overset{nb_{imp}}{\underset{k=1}{\sum }}{\left({\hat{\theta }}_{k}-\hat{\theta }\right)}^{2},$$

$$U=\frac{1}{nb_{imp}(nb_{rep}-1)}\overset{nb_{imp}}{\underset{k=1}{\sum }}\overset{nb_{rep}}{\underset{j=1}{\sum }}{\left({\hat{\theta }}_{k,j}-{\hat{\theta }}_{k}\right)}^{2}.$$

In the above formula, $\tilde{W}$ is the average within imputation variance of the treatment effect estimate, $\tilde{B}$ is the between imputation variance of the average treatment effect estimate across parameter draws and U is the variability of the treatment effect within parameter draws but across imputed datasets. When $\tilde{W}$ and $\tilde{B}$ gets close to W and B, that is, for $nb_{imp}$ and $nb_{rep}$ big enough, the variance formula above includes a new positive term compared to Rubin's rules, U, which is subtracted, leading to a smaller estimated variance and hence narrower confidence intervals. In this two‐stage approach, because we are imputing $nb_{rep}$ times for each parameter draw generated from the imputation model, the successive matching will lead to $nb_{rep}$ matched samples for each parameter draw, thus increasing the probability of a patient being included in one of the final analyses.

Informally, the second term in Rubin's rules accounts for the additional variance introduced due to uncertainty about the missing values. This is estimated by the empirical variance across treatment effects estimated from the different sets of imputed values. However, in the case of propensity score matching, treatment effects resulting from these different sets of imputed values differ not just due to uncertainty about the missing values but also because of the stochastic nature of the sampling process (ie, the propensity score matching). The latter is accounted for within the second term ($\tilde{B}$), but is also accounted for in the original within‐sample variance estimate ($\tilde{W}$), thus we need to subtract an estimate of the additional variability induced by the propensity score sampling process within a fixed dataset (U).

In practice, implementation of these rules, which we will refer to as *Reiter's rules* as a parallel to Rubin's rules, requires a slight modification of the standard implementation of multiple imputation by chained equation: for each parameter draw, $nb_{imp}$ imputed datasets are created instead of only one. This can generally be done using existing options of standard packages for multiple imputation. For example, the ignore argument of the r function mice allows the imputation model to be fitted on a subset of the whole dataset. Therefore, if we concatenate $nb_{rep}$ duplicates of the initial dataset and fix the ignore argument to TRUE for all duplicates, except the first one, this will impute all duplicates using an imputation model based on the same parameter draw estimated from the initial dataset only. This procedure can be repeated $nb_{imp}$ times so that we obtain $nb_{imp}\times nb_{rep}$ imputed datasets, based on $nb_{imp}$ parameter draws sampling with for each, $nb_{rep}$ imputation.

## 3 SIMULATIONS

This section presents results from a simulation study we conducted following the ADEMP framework proposed by Morris et al.^39^

### 3.1 Aims

The aim of the simulation study presented in this section is first to establish whether the discarding of patients in propensity score matching following multiple imputation leads to over‐coverage when Rubin's rules are applied and second to apply Reiter's multiple imputation combination rules (Reiter's rules) to our context and evaluate how they perform.

The number of discarded patients in the matching procedure increases as the number of treated patients decreases (under a 1:1 matching strategy as described below). If discarding patients leads to over‐coverage using Rubin's rules, then we would expect to see larger amounts of over‐coverage as the proportion of patients who are treated decreases. Hence we will simulate scenarios with differing numbers of treated patients to explore whether we observe this phenomenon.

The inverse probability of treatment weighting (IPTW)^20^ approach does not lead to patients being discarded, hence the combination of IPTW and multiple imputation should not suffer from this particular source of potential over‐coverage. Therefore, a final aim of the simulation study is to establish that any over‐coverage seen when combining multiple imputation with Rubin's rules and propensity score matching is not observed when combining the same imputation process with IPTW.

### 3.2 Data generation

We generated $N_{sim}=1000$ datasets, each with N=10000 patients. Three confounders $x=(x_{1},x_{2},x_{3})$ were generated as three independent standard Gaussian variables 𝒩(0,1). Three levels of confounding (strong, moderate and weak) were considered. We expect the crude estimation of the treatment effect to be biased for both the strong and moderate confounding scenarios while we expect the crude estimation to be almost unbiased for the weak confounding scenario.

#### 3.2.1 Treatment and outcome models

The treatment allocation variable z was generated as a Bernoulli variable whose individual probability $\pi_{i}^{T}$ depends upon the three confounders x through a logistic model:

$$\text{logit}(\pi_{i}^{T})=\beta_{0}+\beta_{1}x_{i,1}+\beta_{2}x_{i,2}+\beta_{3}x_{i,3},$$

where the intercept $\beta_{0}$ was chosen so that either approximately 30% ($\beta_{0}=-1$), 20% ($\beta_{0}=-1.4$) or 10% ($\beta_{0}=-2.2$) of the patients were treated.

The outcome variable y was generated as a Bernoulli variable whose individual probability $\pi_{i}^{O}$ depends upon the three confounders x and treatment z through a logistic model:

$$\text{logit}(\pi_{i}^{O})=-1+\gamma_{1}x_{i,1}+\gamma_{2}x_{i,2}+\gamma_{3}x_{i,3}+\theta z_{i}.$$

The three levels of confounding (strong, moderate and weak) were determined according to the values of the model parameters:

- **Strong** confounding: $\beta_{1}=-0.5$; $\beta_{2}=-0.4$; $\beta_{3}=-0.7$; $\gamma_{1}=0.4$; $\gamma_{2}=0.5$; $\gamma_{3}=0.9$; θ=1.2.
- **Moderate** confounding: $\beta_{1}=-0.3$; $\beta_{2}=-0.4$; $\beta_{3}=-0.3$; $\gamma_{1}=0.4$; $\gamma_{2}=0.5$; $\gamma_{3}=0.3$; θ=1.2.
- **Weak** confounding: $\beta_{1}=-0.01$; $\beta_{2}=-0.05$; $\beta_{3}=0.01$; $\gamma_{1}=0.1$; $\gamma_{2}=0.1$; $\gamma_{3}=-0.1$; θ=3.

#### 3.2.2 Missing data model

Only the variable $x_{2}$ was partially missing. For this variable, we considered a missing data scenario with a missing at random mechanism. We simulated a missing data indicator as a Bernouilli variable whose parameter $\pi_{i}^{M}$ follows a logistic regression model

$$\text{logit}(\pi_{i}^{M})=-2+0.1x_{i,1}+x_{i,3}+1.1z_{i}.$$

This model results in approximately 15% of the values of $x_{2}$ being missing.

### 3.3 Estimand

In their most commonly applied forms, propensity score matching estimates the ATT while IPTW estimates the ATE. The true values of these estimands were obtained numerically. We quantify the treatment effect for the ATT and ATE both as an odds ratio and a risk difference. The true ATT values were numerically approximated in the following way. For each scenario, we simulated a sample with a million patients keeping only the treated patients. For those, we saved the values of their outcome and generated a new outcome as if they were untreated, that is, by fixing $z_{i}$ to 0 in the outcome model. Therefore, we have both the potential outcomes for all treated patients in the sample. From that, we can obtain the true ATT values to a high degree of precision. A similar process was followed to obtain the true values of the ATE estimands.

### 3.4 Methods

#### 3.4.1 Assessing confounding in our simulated scenarios

First, we evaluated the level of confounding generated in our simulation scenarios. To this end, we simulated a sample of N=10,000 patients with a binary outcome, without any missing data and with 30% treated (ie, with $\beta_{0}=-1$). For each confounding scenario (weak, moderate and strong) we looked at the balance of the three confounders before matching (in the whole dataset) and after matching (in the propensity score matched dataset). We also looked at the absolute standardized mean differences (ASMD) for all three confounders before and after matching. The ASMD is a balance indicator that helps to identify confounding. Guidelines suggest that ASMD values above 0.1 indicate potential confounding.^40^ To plot balance and ASMD, we used the cobalt package.^41^ Finally, using the same simulated data, the crude treatment effect estimates for the three levels of confounding were compared to the true values to assess the impact of confounding in our simulated scenarios.

#### 3.4.2 Combining multiple imputation and propensity score matching

For all simulations, we set $nb_{imp}=20$ and $nb_{rep}=10$. Simulations were conducted using R. First, multiple imputation was performed using the function mice with the outcome included in the imputation model as advised by previous work^20^, ^42^ to generate $nb_{imp}$ imputed datasets. In the case of Reiter's rules, $nb_{imp}\times nb_{rep}$ imputed datasets were produced and we used the ignore argument of the mice function to generate $nb_{rep}$ imputed datasets for each $nb_{imp}$ parameter draws. Then, for each completed dataset, the propensity score was estimated using the glm function with a logit link including $x_{1}$, $x_{2}$ and $x_{3}$ as covariates. For each completed dataset, each treated patient was matched to one untreated patient by their estimated propensity score, using a caliper of 0.2 times the standard deviation of the logit of the propensity score.^14^ Matching was performed without replacement. This matching was done using the package MatchIt.^43^ For each matched dataset, the ATT was estimated with a generalized linear model of outcome on treatment only, as a risk difference between the treated and the untreated by using a linear link and as an odds ratio between the treated and the untreated by using a logit link. As advised by Austin^7^ and Hill,^18^ clustered standard errors that take into account the within‐pair correlation due to the matched nature of the data were used. In R, this was done using the function glm.cluster from the package miceadds. Finally, all treatment effect estimates and estimation of their variance were aggregated using Rubin's rules or Reiter's rules.

#### 3.4.3 Combining multiple imputation and IPTW

For each of the $nb_{imp}$ imputed datasets, we estimated the ATE using the IPTW approach, aggregating estimates using Rubin's rules. Here, we use the IPTW approach only as a *control* to assess the impact of *not* discarding any patient between multiple imputation and propensity score analysis.

### 3.5 Performance measures

The simulations were evaluated using the following metrics: the relative bias (Rel. bias) defined as the ratio of the absolute bias over the true value and the coverage rate of the 95% confidence intervals.

### 3.6 Results

#### 3.6.1 Confounding in the simulated scenarios

**FIGURE 1:**
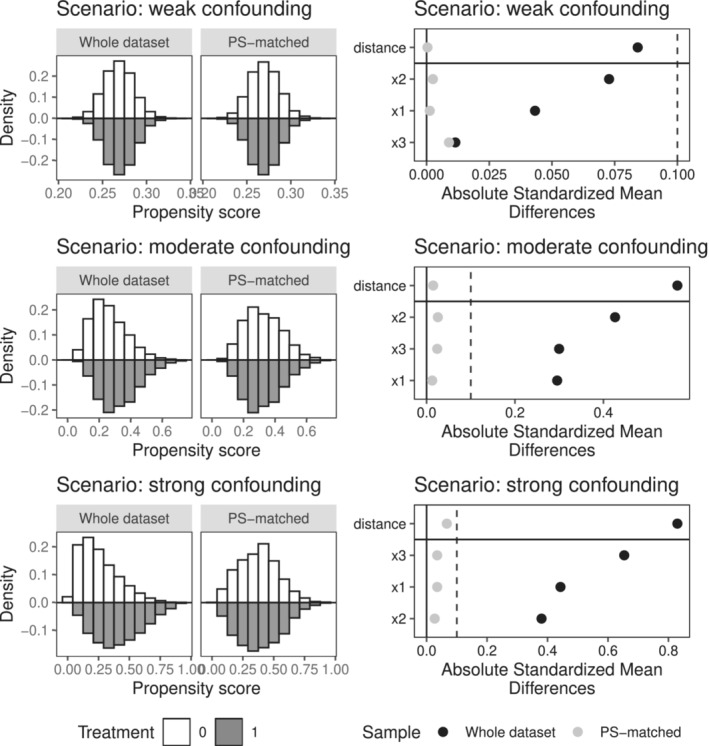
Balance of the distribution of estimated propensity scores between treated and untreated patients before and after matching for all three confounding scenarios: weak, moderate and strong (on the left, from top to bottom). Measures of covariate balance across treatment groups (absolute standardized mean differences) for all three confounders before and after matching for all three confounding scenarios: weak, moderate and strong (on the right, from top to bottom). These plots were obtained using the cobalt package on a simulated sample of N=10,000 patients with a binary outcome, without any missing data and with 30% of treated (ie, with $\beta_{0}=-1$)

Balance and ASMD for the three levels of confounding are displayed in Figure 1. Propensity score distributions among the treated and untreated are very similar in the weak scenario but increasingly different in the moderate and strong scenarios. Little covariate imbalance is observed in the weak scenario (ASMD < 0.01) but large covariate imbalances (ASMD above 0.25) are observed for the moderate and strong scenarios. Overall, we see little potential for confounding in the weak scenario but much stronger in the other two. This is reflected in the unadjusted odds ratio for treatment: with an estimate of 3 in the weak scenario (true value 3) and 0.69 and 0.18 in the moderate and strong scenarios (true value 1.2 in both these settings), indicating strong confounding.

#### 3.6.2 Results when combining multiple imputation and propensity score matching

**FIGURE 2:**
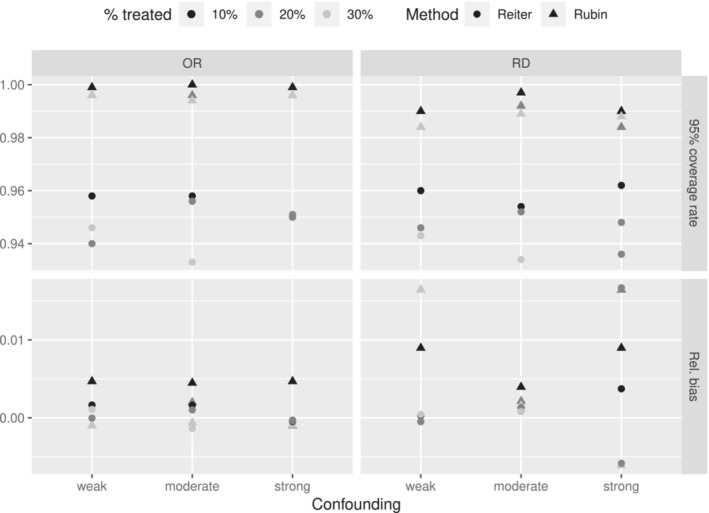
Simulation performance results for a binary outcome for three levels of confounding (weak, moderate, strong), and three levels of treatment percentage (10%, 20%, 30%) on the odds ratio and mean difference scale (ATT) using Rubin's rules and Reiter's rules after applying multiple imputation and then propensity score matching

Relative bias and coverage of the 95% confidence intervals are shown in Figure 2 when Rubin's rules and Reiter's rules are applied after applying multiple imputation and then propensity score matching under the three levels of confounding and three levels of percentage of the sample treated. The ATT is quantified both as an odds ratio (left) and a risk difference (right).

From Figure 2, we can see that both Rubin's rules and Reiter's rules give approximately unbiased point estimates with relative bias very close to 0. Application of Rubin's rules led to higher than nominal coverage, around 0.99. This over‐coverage is a consequence of a general discrepancy between the empirical and the model standard errors, the latter being systematically bigger than the former. As we hypothesized, this over‐coverage of the confidence interval increases when the percentage of treated patients decreases, that is, when more patients are discarded between the imputation and the treatment effect estimation.

Conversely, Figure 2 shows that using Reiter's rules led to coverage rates much closer to the nominal value of 0.95.

#### 3.6.3 Results when combining multiple imputation and IPTW

**FIGURE 3:**
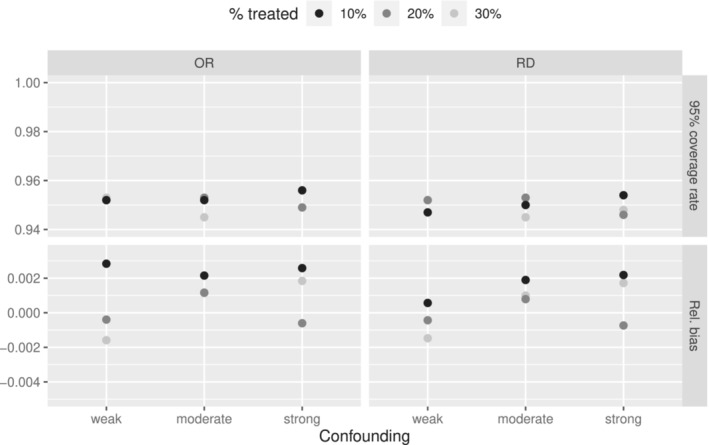
Simulation performance results for a binary outcome for three levels of confounding (weak, moderate, strong), and three levels of treatment percentage (10%, 20%, 30%) on the odds ratio and mean difference scale (ATE) using Rubin's rules after combining multiple imputation and IPTW

Relative bias and coverage of the 95% confidence intervals are shown in Figure 3 when Rubin's rules are used after applying multiple imputation and then IPTW under the three levels of confounding and three levels of percentage of the sample treated. The ATE is quantified both as an odds ratio (left) and a risk difference (right).

From Figure 3, we observe that the combination of multiple imputation and IPTW leads to unbiased estimate of the ATE and the coverage rates are close to the nominal value of 0.95 when using Rubin's rules as aggregator of the estimates. This was expected because, in contrast to propensity score matching, IPTW does not discard any patients so the set of patients used in the imputation step match the set used in the treatment effect estimation step.

## 4 APPLICATION

In this section, we use data taken from the National Cancer Registry of the Office for National Statistics^44^ to estimate the effect of age at diagnosis as a binary variable (using the median as the cutoff) on the receipt of surgery for the 31,351 patients diagnosed with lung cancer recorded in the registry. Tumor stage at diagnosis is classed as early versus late, based on a dichotomization (stages 1,2 vs 3,4) of Belot et al's algorithm.^44^ The patient's performance status, assessing functional abilities, has two modalities, good and bad, based upon dichotomization of the five‐category WHO classification.^44^ Deprivation was measured using the Income Domain from the 2010 England Indices of Multiple Deprivation.^45^ Comorbidities were adjusted for using the Charlson Comorbidity Index with a 6‐year time window up to 6 months before diagnosis. All these variables and the sex of the patient were considered to be confounders in our analysis. Table 1 provides a description of the sample by the outcome, receipt of surgery, summarizing potential confounder variables and any missing data. About 25% of performance status and 10% of tumor stage data were missing.

**TABLE 1:** Descriptive statistics and missing data summary for potential confounders used in our illustrative example from the National Cancer Registry dataset according to the outcome: absence or presence of surgery

	No surgery received	Surgery received	Overall
Surgery	(N = 26 501)	(N = 4850)	(N = 31 351)
Age (binary)			
< median	12 371 (46.7%)	3304 (68.1%)	15 675 (50.0%)
> median	14 130 (53.3%)	1546 (31.9%)	15 676 (50.0%)
Sex			
Male	14 729 (55.6%)	2463 (50.8%)	17 192 (54.8%)
Female	11 772 (44.4%)	2387 (49.2%)	14 159 (45.2%)
Stage			
Early	3339 (12.6%)	3823 (78.8%)	7162 (22.8%)
Late	20 488 (77.3%)	878 (18.1%)	21 366 (68.2%)
Missing	2674 (10.1%)	149 (3.1%)	2823 (9.0%)
Performance status			
Good	9332 (35.2%)	3728 (76.9%)	3728 (76.9%)
Bad	10 309 (38.9%)	340 (7.0%)	10 649 (34.0%)
Missing	6860 (25.9%)	782 (16.1%)	7642 (24.4%)
Deprivation			
Mean (SD)	0.690 (0.463)	0.653 (0.476)	0.684 (0.465)
Charlson score			
Mean (SD)	1.34 (1.66)	1.04 (1.32)	1.29 (1.62)

**FIGURE 4:**
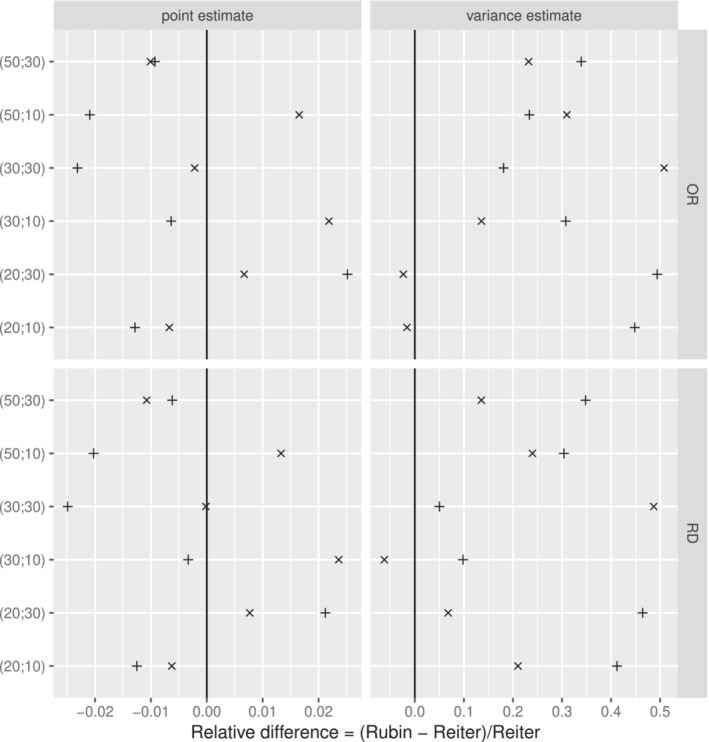
Relative differences for the point estimates (left pane) and the estimated variances (right pane) between Rubin's rules and Reiter's rules in the OR scale (upper pane) and RD scale (lower pane) using both random seeds (✛ for 1604 and ✕ for 1993) and for each value of $\left(nb_{imp};nb_{rep}\right)$

Multiple imputation was performed using mice, by including the binary outcome, treatment and the fully observed confounders listed above in the imputation model. Propensity score matching was performed using nearest neighbor matching without resampling. A caliper of 0.2 was applied on the scale of the logit of the propensity score values. We applied the procedure described in Section 3.4.2 to these data using both Rubin's rules and Reiter's rules. In order to assess the impact of the choice of $nb_{imp}$ and $nb_{rep}$, several combinations of values were used: $\left(nb_{imp};nb_{rep})=(20;10\right)$, (20;30), (30;10), (30;30), (50;10) and (50;30). We also used two different seeds, 1604 and 1993, to assess the potential impact of random fluctuation on the results. For each scenario, the treatment effect was quantified using both the odds ratio and the risk difference, with the point estimate and the estimated variance obtained using both Rubin's rules and Reiter's rules. We computed the relative difference between Rubin's and Reiter's rules point estimates and variances defined as the difference between Rubin's and Reiter's rules point estimates and variances over the Reiter's rules point estimate and variance. This allows the comparison of the two approaches in a more standardized way than just looking at the raw differences. Figure 4 shows these relative difference for all scenario considered.

For both Rubin's rules and Reiter's rules, the point estimate is obtained as the mean of estimated treatment effects across imputed datasets. Therefore, on average we would expect the difference in point estimates to be zero. Figure 4 shows, as expected, that the relative difference in point estimates is scattered around zero, demonstrating no systematic difference between the two approaches. The variance estimate obtained from Reiter's rules is expected to be smaller than that from Rubin's rules (although not mathematically guaranteed to be smaller), therefore we would expect the relative difference to be positive in general. Indeed, in almost all scenarios the relative difference is positive, meaning that the estimated variance is systematically smaller when using Reiter's rules rather than Reiter's rules. For the three remaining scenarios, the variance was only slightly smaller using Rubin's rules, compared to using Reiter's rules. Also, these three cases happen only in scenarios where values of $\left(nb_{imp};nb_{rep}\right)$ are low. We would expect that increasing these numbers would result in $\tilde{W}$ and $\tilde{B}$ becoming closer to W and B, resulting in the variance from using Reiter's rules being at least as small as that using Rubin's rules.

Table 2 displays the point estimates and 95% confidence intervals of the ATT on the risk difference scale using Rubin's and Reiter's rules, using both random seeds and for the different values of $\left(nb_{imp};nb_{rep}\right)$. From this Table, it is clear that Reiter's rules lead to narrower confidence intervals. Also, it seems that, assuming $nb_{imp}$ constant, increasing $nb_{rep}$ lead to narrower confidence intervals in most of the cases for Reiter's rules results. Increasing, $nb_{imp}$ from 20 to 30 also lead to narrower confidence intervals for both Rubin's rules and Reiter's rules but increasing it to 50 does not lead to a substantial higher precision.

**TABLE 2:** Point estimate and 95% confidence intervals for the ATT on the risk difference scale using Rubin's and Reiter's rules, using two different random seeds and a range of values for ($nb_{imp},nb_{rep}$)

	Rubin's rules		Reiter's rules		
$nb_{imp}$	Seed 1993	Seed 1604	$nb_{rep}$	Seed 1993	Seed 1604
20	−0.575 (−0.580; −0.570)	−0.568(−0.575; −0.562)	10	−0.579 (−0.584; −0.573)	−0.576 (−0.579; −0.572)
			30	−0.571 (−0.576; −0.566)	−0.554 (−0.557; −0.551)
30	−0.578 (−0.585; −0.071)	−0.562 (−0.568; −0.556)	10	−0.565 (−0.571; −0.559)	−0.556 (−0.570; −0.562)
			30	−0.579 (−0.583; −0.575)	−0.575 (−0.580; −0.570)
50	−0.578 (−0.584; −0.572)	−0.564 (−0.569; −0.559)	10	−0.568 (−0.572; −0.564)	−0.576 (−0.580; −0.572)
			30	−0.584 (−0.588; −0.579)	−0.569 (−0.573; −0.566)

In this application, when $\left(nb_{imp};nb_{rep}\right)$ goes from (20,10) to (20,30), the computational time was multiplied by 3; when it goes from (20,10) to (30,10) it was multiplied by 1.4. As we would expect, the Monte Carlo error is reduced by increasing both $nb_{imp}$ and $nb_{rep}$. However, computational burden may limit the feasibility of using very high numbers, in which case, we suggest re‐running the analysis using a different seed to assess the robustness of results.

## 5 DISCUSSION

In this article, we demonstrated that estimating a treatment effect using propensity score matching, after using multiple imputation to handle missing data, leads to over‐coverage in the confidence interval for the treatment effect, when Rubin's rules are used to estimate the variance of the treatment effect estimate. This over‐coverage is due to using data in the imputation from patients whose data is not used in the subsequent estimation of the treatment effect. We demonstrated that Reiter's correction^21^ to Rubin's rules, introduced to solve a related problem in a different context, removed this over‐coverage in a range of simulation settings.

In a recent simulation study^37^ over‐coverage was also observed when combining multiple imputation and propensity score matching using Rubin's rules. However, because inflated standard errors were observed in the absence of missing data, the authors attributed the over‐coverage to the standard error estimator being conservative, rather than being a consequence of applying multiple imputation in this setting.

Reiter's rules have the advantage of being easy to implement using the R package mice and its ignore argument as we detailed on Section 2. A drawback of Reiter's rules, however, is that when doing $nb_{imp}$ imputations for each of the $nb_{rep}$ parameter draws this leads to a total of $nb_{imp}\times nb_{rep}$ imputed datasets, requiring the process of propensity score matching and treatment effect estimation to be repeated $nb_{imp}\times nb_{rep}$ times. The choice of $\left(nb_{imp};nb_{rep}\right)$ when using Reiter's rules is therefore important and is a compromise between the computational burden and the precision of the method. When working with big sample sizes, implementing Reiter's rules may become computationally burdensome. However, in many standard situations with modest sample sizes, this is not an issue and Reiter's rules can be easily applied.

In this article, we have focused on the propensity score matching approach only because the issue of inflated variance only arises with this propensity score method. This is because among the various propensity score approaches, matching is the only one which discards a large portion of patients from the initial dataset leading to an inconsistency between the sample used to impute the missing data and the one used to estimate the treatment effect. In our simulation settings, using the IPTW approach to estimate the average treatment effect using Rubin's rules to compute the variance results in coverage rates close to the nominal value, consistent with results from previous work.^20^, ^37^

We have focused on binary outcomes in this article. In principle, the same phenomenon of over‐coverage is likely to arise when combining multiple imputation with Rubin's rules and propensity score matching. However, with a continuous outcome, obtaining a standard error that correctly accounts for all sources of variability—including the estimation of the propensity score—in the absence of missing data is more challenging. This makes it hard to clearly disentangle incorrect coverage due to lack of correction for the propensity score estimation with that due to the phenomenon explored in the current article.

While we explored one particular variance estimator in our simulation studies, we expect the over‐coverage identified to occur when using other variance estimators. We note that different estimators do not always account for the same sources of variability in the full data (eg, some account for the estimation of the propensity score and some do not), which would impact their relative performance with or without missing data. We have therefore avoided this additional complicating factor by focusing on one variance estimator only.

In this paper, we have explored only a small number of simulation settings. We identified the over‐coverage we expected to find and showed that, in these situations, Reiter's correction removed the over‐coverage, as expected. More research is needed to explore this phenomenon in different settings, and to develop guidance on how to optimally choose the numbers of different phases of imputations ($nb_{i}mp$ and $nb_{r}ep$).

## Data Availability

Data sharing is not applicable to this article as no new data were created or analyzed in this study.
